# Prevalence and predictors of primary postpartum hemorrhage: An implication for designing effective intervention at selected hospitals, Southern Ethiopia

**DOI:** 10.1371/journal.pone.0224579

**Published:** 2019-10-31

**Authors:** Biruk Assefa Kebede, Ritbano Ahmed Abdo, Abebe Alemu Anshebo, Beminet Moges Gebremariam

**Affiliations:** 1 Department of Midwifery, College of Medicine and Health Sciences, Wachemo University, Hossana, Ethiopia; 2 Department of Public Health Officer, College of Medicine and Health Sciences, Wachemo University, Hossana, Ethiopia; Ospedale dei Bambini Vittore Buzzi, ITALY

## Abstract

**Background:**

Primary postpartum hemorrhage is the leading cause of maternal mortality worldwide. Ethiopia has made significant progress in maternal health care services. Despite this, primary postpartum hemorrhage continues to remain the leading cause of maternal mortality in Ethiopia. This study aimed to assess the prevalence and predictors of primary postpartum hemorrhage among mothers who gave birth at selected hospitals in the Southern Ethiopia.

**Methods:**

An institution-based cross-sectional study was employed from March 2–28, 2018. Four hundred and twenty-two study participants were obtained using the consecutive sampling method. A structured interviewer-administered questionnaire and chart review were used to collect data. Data were entered into Epi-data version 3.1 and analyzed using SPSS version 22. Multivariable logistic regression were used to determine the predictors of primary postpartum hemorrhage with 95% CI and p-value < 0.05.

**Results:**

The overall prevalence of primary postpartum hemorrhage was 16.6%. Mothers aged 35 and above [AOR = 6.8, 95% CI (3.6, 16.0)], pre-partum anemia [AOR = 5.3, 95% CI (2.2, 12.8)], complications during labor [AOR = 1.8, 95% CI (2.8, 4.2)], history of previous postpartum hemorrhage [AOR = 2.7, 95% CI (1.1, 6.8)] and instrumental delivery [AOR = 5.3, 95% CI (2.2, 12.8)] were significant predictors of primary postpartum hemorrhage.

**Conclusion:**

Primary postpartum hemorrhage is quite common in the study area. Mothers aged 35 and above, complications during labor, history of previous postpartum hemorrhage, and instrumental delivery were predictors of primary postpartum hemorrhage. Since postpartum hemorrhage being relatively common, all obstetrics unit members should be prepared to manage mothers who experience it.

## Introduction

Primary postpartum hemorrhage (PPH) is defined as a cumulative blood loss ≥ 500ml following vaginal delivery or ≥1000ml following cesarean delivery or any amount of blood loss within 24hours after birth evidenced by a rise in pulse rate, and falling blood pressure [1]. It is the leading cause of maternal mortality, accounting for about 19.7% all pregnancy related deaths worldwide. The rates of maternal death associated with PPH are highest in the countries with low income and middle-income regions and accounted for 480 000 (32%) of deaths in the northern Africa, but only for 1200 (8%) in the developed regions [2].

The prevalence of PPH has been reported 1.2% in multicountry Survey on Maternal and Newborn Health and are estimated as substantially higher in the developing countries [3]. In contrast, recent studies have shown an increase in the incidence of primary postpartum hemorrhage in the developed countries as well [4–6].

Atonicity of the uterus is the commonest cause of PPH: with the separation of the placenta, the uterine sinuses, which are torn, cannot be compressed effectively due to imperfect contraction and retraction of the uterine musculature and the bleeding continues[1, 4, 7–11]. However, a studies conducted in Nigeria [12], Denmark [13], and Ethiopia [14] revealed the commonest causes of postpartum hemorrhage were genital trauma and retained placenta.

Primary postpartum hemorrhage may develop in patients with no risk factors [15, 16]. However, there have been a number of previous studies attempting to identify predictors of postpartum hemorrhage in different countries include previous PPH [7, 17], multiple pregnancy [17, 18], pre-partum anemia [5, 11, 19], large baby [18, 19], placenta praevia [20, 21], induction of labor [22, 23], prolonged labor [10, 23, 24], operative vaginal delivery [5, 19], delivery by Caesarean section [18, 22], preeclampsia [10, 17], mothers age 35 or above years [18, 26], multiparity [23, 24, 26], post term pregnancy[3] and chorioamnionitis[6].

Comparing with PPH, mothers face to higher risk of several complications, including severe anemia, hepatic failure, Acute Respiratory Distress Syndrome, need a blood transfusions, open surgery, care in intensive care units, disseminated intravascular coagulation, hysterectomy and cardiac arrest [8, 15, 27]. In mild cases, PPH can lead to mild anemia, fatigue, depression and feelings of separation or anxiety [28, 29].

Ethiopia has made significant progress in maternal health care services include the increases institutional births, avail skilled birth attendants at all births and practicing active management of the third stage of labor which reduces the incidence of PPH, the quantity of blood loss and the need for blood transfusions [30]. Despite this, PPH continues to remain the leading cause of maternal mortality in Ethiopia [31–34].

Estimation of PPH is required to implement interventions to reduce the risk of maternal death and morbidity. So, the main objective of this study was to assess the prevalence and predictors of PPH among mothers who gave birth at selected hospitals in the Southern Ethiopia. In that way, the results of the study will help policy-makers, program designers and Non-governmental organizations to support the study area.

## Methods and materials

It was an institution-based cross-sectional conducted in the Wachemo University Negist Eleni Mohammed Memorial General Hospital, Butajira Zonal Hospital and in the Worabe comprehensive Hospital from March 2–28, 2018. Source populations were all mothers who gave birth in the selected Hospitals during the study period and mothers who cannot able to communicator critically ill/sick at the time of data collection were excluded from the study.

The single population proportion formula was used to determine sample size with the following assumptions: prevalence of primary postpartum hemorrhage was 50%, 95% confidence interval, marginal error 5% and 10% none response rate, the final sample size was 422. Three hospitals were selected purposely. From all hospitals, 422 participants were selected using a consecutive sampling technique till the calculated sample size was achieved. The allocation of the study participants to each hospital was based on the previous monthly deliveries (from hospital records). Data were collected using a pretested structured interviewer administered questionnaire and patient's chart reviewed, which was used to retrieve diagnosis of primary postpartum hemorrhage and mothers’ test results that could not be captured by the interview. Research questionnaire was developed based on the instruments that were applied in other related studies [11, 14, 24–26]. It was intended to collect data on sociodemographic variables, obstetric related characteristics (antepartum, intrapartum and postpartum events) and fetal factors. Three B.Sc. midwives, and one M.Sc. midwife were recruited for the data collection and supervision, respectively in each hospital.

To ensure the quality of data collected from the study participants, at the beginning, a data collection questionnaire was pre-tested on 5%(21) of calculated sample size at the Halaba Zonal Hospital and necessary modifications were made based on gaps identified in the questionnaire. Data collectors and supervisors were trained for three days intensively on the study instrument and data collection procedure that includes the relevance of the study. The English questionnaire was translated first to the local language and translated back into English language by experts to check its consistency. The data collectors worked under close observation of the supervisors to ensure reliability to correct data collection procedures. In addition, supervisors and the principal investigators checked the filled questionnaires at the end of data collection every day for completeness. Furthermore, the data were carefully entered and cleaned before the beginning of the analysis.

### Measurements

#### Primary postpartum hemorrhage

In this study the definition of clinical diagnosis PPH was obtained from the mothers’ card which was identified by birth attendants and was classified as: “yes” (having a clinical diagnosis of postpartum hemorrhage in the mothers’ card) or “no”.

#### Complications during Pregnancy

Such as antepartum hemorrhage, hypertension disorders during pregnancy, polyhydramnios, chorioamnionitis or/and others.

#### Complications during Labor

Malpresentation, malposition, prolonged labour or obstructed labour, or/and others, (present = 1 or absent = 0).

### Data analysis

Data were entered using Epi-data version 3.1 and exported to statistical package social science (SPSS), version 22.0 software for analyses. Multivariable logistic regression was done for variables that have p-value ≤ 0.25 during the bivariate logistic regression analyses to identify the predictor of PPH and to control for potential confounders. The degree of association between independent, and dependent variables were assessed using odds ratio with 95% confidence interval. The P-value < 0.05 was considered as statistically significant. The Hosmer-Lemeshow goodness-of-fit statistic was used to check if the necessary assumptions for multivariable logistic regressions were fulfilled and the model had p-value > 0.05 which proved the model was good.

### Ethics approval and consent to participation

Ethical approval was taken from the Institutional Review committee of Wachemo University. Formal letters were obtained from the Hadiya, Silte and Gurage zonal health office administration. Then, permission was obtained from each hospital authority before commencing the data collection. The participants were informed about purpose, procedures, potential risks and benefits of the study. Informed written consent was sought from selected participant to confirm willingness to participate in the study before the interview. To protect confidentiality, name was not included in the written questionnaire. The study participants also were ensured that refusal to consent or withdrawal from the study would not alter or put at risk their access to care.

## Results

### Sociodemographic characteristics

A total of 422 mothers were involved in this study making the response rate 100%. About 83.9% mothers were aged 20–34 years, the range between 18–40 years with a mean (±SD) 27.44 (±4. 8) years. The majority of mothers were married 417 (98.8%), 316 (74.9%) had completed primary school and 257 (60.9%) were housewife **(Table 1**).

**Table 1:** Socio- demographic characteristics of study participants at selected hospitals, Southern Ethiopia, 2018.

Variables	Frequency(N = 422)	Percent
**Age group**		
≤20	23	5.5
20–34	354	83.9
≥35	45	10.7
**Marital status**		
Married	417	98.8
Others	5	1.2
**Women's education**		
Completed 1–8 and below	316	74.9
High and preparatory school completed	69	16.4
Higher institution	37	8.8
**Women’s occupation**		
House wife	257	60.9
Merchant	61	14.5
Government employee	93	22.0
Others*	11	2.6
**Average monthly income in Ethiopian birr**		
<1000	241	57.1
1001–2000 2001–3000	76	18.0
	87	20.6
>3000	18	4.3

* = other include self-employed, daily laborer and student

### Obstetric related characteristics of the study participants

Regarding their gravidity, 100 (23.7%) mothers were grand multipara. The majority (93.4%) of mothers had a history of antenatal care follow-up. The study showed that forty one (9.7%) mother's encountered postpartum hemorrhage in previous delivery and thirty mothers (7.1%) had a history of stillbirth.

The study showed that sixty one (16.1%) mothers encountered complications during pregnancy among which the leading cause was pregnancy induced hypertension 26 (38.2%) followed by antepartum hemorrhage 16 (23.5%). Among all deliveries attended, 52 (12.3%) had experienced complications, of which prolonged labor accounting 28 (6.6%), followed by the imposition/ malpresentation 26 (6.2%) and thirty-one (9%) women had ever experienced abortion. Regarding the mode of delivery, about 345 (81.8%) were normal vaginal delivery and fifty seven (22.3%) of mothers had a pre-partum anemia **(Table 2).**

**Table 2:** Obstetric history of the study participants, at selected hospitals, Southern Ethiopia, 2018.

Variable	Frequency(N = 422)	Percentage
**Gravidity (N = 422)**		
Primigravida	76	18.0
Multigravida^a^	246	58.3
Grand multigravida^b^	100	23.7
**Antenatal Care follow up(N = 422)**		
Yes	400	94.8
No	22	5.2
**Ever had abortion (N = 346)**		
Yes	31	9.0
No	315	91.0
**History of previous caesarean section**		
Yes	16	3.8
No	406	96.2
**History of previous primary postpartum hemorrhage**		
Yes	41	9.7
No	381	90.3
**Previous still birth**		
Yes	30	7.1
No	392	92.9
**Current pregnancy complications**		
Yes	68	16.1
No	355	83.9
Types of complication**		
Antepartum hemorrhage	16	23.5
Premature rupture of fetal membranes	14	20.6
Pregnancy induced hypertension	26	38.2
Others *	12	17.6
**Status of labor(n = 422)**		
Spontaneous	415	98.3
Induced	7	1.7
**Complications of labor(n = 422)**		
Yes	52	12.3
No	370	87.7
Type of labor complication**		
Prolonged Labor	**28**	6.6
Malposition/presentation	26	6.2
Obstructed Labor	7	1.7
**Mode of delivery(n = 422)**		
Spontaneous vaginal delivery	345	81.8
Cesarean section	35	8.3
Instrumental	42	10.0
**Pre-partum anemia**		
No	365	86.5
Yes	57	13.5

*Others include hyperemesis gravidarum, and polyhydramnios

** indicate that it is not taken out of 100%.

^a^ A woman who has had two or more pregnancies

^b^ The fact of having given birth to more than four children

### Prevalence of primary postpartum hemorrhage and birth outcome

The prevalence of primary postpartum hemorrhage was 16.6% (N = 70). 418 (99.1%) neonates were live births and 4 still births (0.9%). Three hundred and ninety six newborns had a normal birth weight (94.7%) and four hundred seven (96.4%) were born at term **(Table 3)**.

**Table 3:** Prevalence of primary postpartum hemorrhage and birth outcomes among mothers who gave birth at selected hospitals, Southern Ethiopia, 2018.

Variables	Frequency	Percentage
**Primary post-partum hemorrhage (N = 422)**		
No	352	83.4
Yes	70	16.6
**Birth weight of live birth babies (N = 418)**		
Low birth weight (< 2500gram)	7	1.7
Normal birth weight (2500-4000gram)	396	94.7
Large birth weight(>4000gram)	15	3.6
**Gestational age (N = 422)**		
Preterm(<37weeks)	8	1.9
Term(37-42weeks)	407	96.4
Unknown	7	1.7

### Identified causes of primary postpartum hemorrhage

As reported on mothers' card, the commonest cause was atonic uterus 50(71.4%) and followed by genital trauma (14.3%) and retained placenta (14.3%).

### Predictors of primary postpartum hemorrhage

In multivariable logistic regression analysis, mothers aged 35 and above, the presence of any complication during the pregnancy, complication during labor, pre-partum anemia, a history of previous postpartum and instrumental delivery were found to be predictors of PPH.

Mothers aged 35 and above were nearly seven times more likely to have experienced PPH respect to women in the age group between 20–34 years old [AOR = 6. 8; 95%CI (3.6, 16.0)]. The presence of a pregnancy complication was nearly five times more likely to have PPH than their counterparts (AOR = 4.7, 95% CI (2.2, 10.1)). Similarly, mothers who developed a complication during labor were nearly two times more likely to develop PPH [AOR = 1.8; 95% CI (2.8, 4.2)] than to their counterparts. Also, the likelihood of PPH was increased for mothers whose mode of delivery was instrumental vaginal delivery [AOR = 5. 3; 95%CI: 2.2, 12.8]. In addition, mothers with pre-partum anemia was seven or more times more likely to encounter PPH compared to no anemia during pre-partum period [AOR = 7. 4, 95%CI (3.6, 15.3)] **(Table 4).**

**Table 4:** Bivariate and multivariable logistic regression of selected variables in relation to primary postpartum Hemorrhage among mothers who gave birth at selected hospitals, Southern Ethiopia, 2018.

Variables	Primary Postpartum Hemorrhage		COR(95%CI)	AOR (95%CI)
	No	Yes		
**Age group**				
<20	18	5	1.9(.7, 5.3)	1.8(0.7,7.2)
20–34 (ref.)	308	46	1	1
35 and above	26	19	4.9(2.5 9.5)*	6.8(3.6, 16.0)**
**Pregnancy complications**				
No(ref.)	307	48	1	1
Yes	45	22	3.1(1.7, 5.6)*	4.7(2.2, 10.1)**
**History of previous PPH**				
No(ref.)	327	54	1	1
Yes	25	16	3.9(1.9, 7.7)*	2.7(1.1, 6.8)**
**Complications during Labor**				
No(ref.)	316	54	1	1
Yes	36	16	2.6(1.4, 5.0)*	1.8(2.8, 4.2)**
**Pre-partum anemia**				
Yes	30	27	6.7(3.7, 12.4)*	7.4(3.6, 15.3)**
No (ref.)	322	43	1	1
**Mode of delivery**				
Normal vaginal delivery(ref.)	298	47	1	1
Caesarean Section	30	5	1.1(.4, 2.9)	2.3(.8,7.1)
Instrumental	24	18	4.8(2.4, 9.4)*	5.3(2.2,12.8)**

* = p≤.25

** = p < 0.05

COR: Crude odd ratio

AOR: Adjusted odd ratio

## Discussion

The overall prevalence rate of primary postpartum hemorrhage was 16.6%. This prevalence is higher compared to other studies in Japan, India, Uganda, Zimbabwe and Ethiopia, where it is 13%, 3.3%, 9%, 1.6% and 5.8%, respectively [5, 9, 10, 18, 24]. Probably because the study hospitals are referral hospitals of lower level health facilities, but also, this difference could be an indicator of ineffectiveness of the national strategies for maternal health care services.

However, the prevalence rate was lower than reports in Cameroon 23.6% [7], Yemen 29.1% [11] and Pakistan 21.3% [26]. This variation in our study might be due to difference in study design, social stability, cultural difference and maternal health care services accessible. In addition, the prevalence of PPH may vary between and within geographical regions.

In this study, the mothers aged of 35 or above was one of the predictors for PPH. This finding was almost found to be a universal fact, the mother's age increases the risk of obstetric complications including PPH [35–37]. Similar findings were also reported from the studies done in France [6], Uganda [18] and Pakistan [26] which revealed that mothers aged 35 or above was more likely to experience a PPH.

The study showed that mothers with a history of previous postpartum hemorrhage was found at more risk of primary postpartum hemorrhage than those with no history PPH. This finding was similar to previous studies done in Cameroon [7] and Norway [17].

Pre-partum anemia was a found to be predictor of PPH in this Study. Reason for these may be attributed to mother with pre-partum anemia may develop primary postpartum hemorrhage with a minimum amount of blood loss after delivery. It is possible to early identify mothers with anemia in their antenatal care follow-up, and take appropriate measures. This is supported by a researches done in Japan [5], Yemen [11], and Norway [17] and, Senegal and Mali [19].

Primary postpartum hemorrhage was also found to be associated with pregnancy complications in this study. This might be related to coagulation defect due to severe pre-eclampsia and chorioamnionitis which may affect clotting factors, and mothers with a history of antepartum hemorrhage develop primary postpartum hemorrhage with the slight blood loss after delivery. This finding was almost found to be a universal fact and has been revealed in many studies [6, 10, 17, 20].

Complications during labor was found to be predictors of primary postpartum hemorrhage. Similar to what was found in a studies conducted in Cameroon, Zimbabwe, Côte D’Ivoire and Ethiopia [7, 10, 23, 24]. This may be explained by the fact that the study hospitals are referral centers.

As revealed by the present study, high parity was found to have significant association with primary postpartum hemorrhage, which is in line with what has been found in Côte D’Ivoire, Ethiopia and Pakistan [23, 24. 26]. The reasons for this may be due to the fact that repeated stretching of muscle fibers leads to the loss of muscle tone that results in uterine atony.

Instrumental delivery was associated with PPH, similar to what was reported in Japan [5] and Senegal [19]. May be because an instrumental delivery increases the risk for vaginal, cervical, or perineal lacerations.

As reported the main cause of PPH on mothers' chart/card, (N = 50, 71.4%) of primary postpartum hemorrhage cases were due to the atonic uterus. This finding is well known in literature.

This study clearly shares the limitations of cross-sectional studies and there may also be observed variations as different clinicians with differences in their grade, training, and experience made the diagnosis of PPH.

## Conclusion

Primary postpartum hemorrhage is quite common in the study area. Mothers aged 35 and above, complications during labor, history of previous postpartum hemorrhage, and instrumental delivery were determinants of PPH. Since primary postpartum hemorrhage being relatively common, all obstetrics unit members should be prepared to manage mothers who experience it. All health facilities should consider a way to recognition and prevention of measures in place for all mothers. All obstetric units should have guidelines for the routine administration of uterotonics in the immediate postpartum period and practice active third stage management for all mothers.

## Supporting information

S1 Data SPSS. (SAV)

S1 Questionnaire English questionnaire. (DOCX)
