# Supplementary material for: Prevalence and predictors of primary postpartum hemorrhage: An implication for designing effective intervention at selected hospitals, Southern Ethiopia
Source: PLoS One. 2019 Oct 31;14(10):e0224579. doi: 10.1371/journal.pone.0224579 (PMC6822730; doi:10.1371/journal.pone.0224579)
Supplement: S1 Questionnaire — (DOCX) [file pone.0224579.s002.docx]

**Appendix I. Participant Information sheet (English Version)**

**Dear Respondent:**

My name is _____________________. This questionnaire is prepared to assess the Prevalence and Predictors of Primary Postpartum Hemorrhage: An Implication for Designing Effective Intervention at Selected Hospitals, Southern Ethiopia, 2018. You are selected and included in the study as part of the sample population to complete the questionnaire designed by the researchers. Since there is no research done on this topic in the study area, provision of such data on this very important subject may alert researchers, health workers, community and individuals for better health of mothers and newborns. It will be used as reference for those who are interested to perform a research on the same topic. The study will involve you completing the questionnaire that is enclosed with this letter and it will not take more than 20 minutes to complete. Confidentiality and anonymity is fully assured, as your name is not required on the questionnaire and only the research team will have access to the results.

**Informed Consent Form**

Dear my participant, I would be grateful if you would participate in this study by completing this questionnaire. It hoped that the findings of this study would help to identify predictors of Primary Postpartum Hemorrhage: An Implication for Designing Effective Intervention at Selected Hospitals, Southern Ethiopia, 2018. Be assured that the information you provide will be used for research purposes only and will be treated as confidential and participation will be based on voluntarily will**.** I also would like to assure you that any time you feel uncomfortable participating on the study and withdrawing from the study. The study does not have any effect on the service you obtain from the respective health facility.

If you would like to know more, please contact:

**Address of the Principal Investigators Name**: Biruk Assefa, 0910143410

I thank you in advance for taking your time to answer questions.

Do you have any opinion regarding this study?

Do you agree to participate in this study?

Yes, continue No, thank you!

Signature of Participant_____________________ Date_____________

**PART I: SOCIODEMOGRAPHIC CHARACTERSTICS**

| **Code** | **Questions** | **Possible Answers** | **Skip** |
| --- | --- | --- | --- |
| 101 | Age | ____________ years |  |
| 102 | Address | _____________ |  |
| 103 | Marital status | 1. Single 2. Married 3. Divorced 4. Widowed 5. Others |  |
| 104 | Educational status | 1. Illiterate 2. Completed first cycle (1-4) 3. Completed second cycle (5-8) 4. Completed 9-12 5. Attained tertiary education |  |
| 105 | Occupation | 1. government employee 2. Self employed 3. House wife 4. merchant 5. others (specify) |  |
| 106 | Monthly income estimated in birr | _______________ |  |

**Part II. Obstetric History**

| **Code** | **Questions** | **Possible Answers** | **Skip** |
| --- | --- | --- | --- |
| 201 | Gravidity/ Parity | _____/________ |  |
| 202 | Gestational age (weeks) | 1. _________wks 2. Unknown |  |
| 203 | ANC (antenatal care) | - - 1. yes     2. No |  |
| 204 | History of abortion | 1. No 2. Yes | If 0, skip to Q206 |
| 205 | If yes, how many times | _________no |  |
| 206 | History of previous C/S | 1. No 2. Yes |  |
| 207 | If yes , how many times | __________ |  |
| 208 | History of previous postpartum Hemorrhage | 1. No 2. Yes |  |
| 209 | Previous still birth | 1. No 2. Yes |  |
| 210 | Previous uterine curettage | 1. No 2. Yes |  |

**Part III. Ante Partum Obstetric Event**

| **Code** | **Questions** | **Possible Answers** | **Skip** |
| --- | --- | --- | --- |
| 301 | Abruption placenta | 1. No 2. Yes |  |
| 302 | Placenta Previa | 1. No 2. Yes |  |
| 303 | Current ante partum hemorrhage | 1. No 2. Yes |  |
| 304 | Polyhydramnios | 1. No 2. Yes |  |
| 305 | Delivery characteristics | 0. Singleton  2. Twins  3. Higher Order |  |
| 306 | Pregnancy induced hypertension(pre-eclampsia, eclampsia …) |  |  |
| 307 | Prepartum Hematocrit/Hemoglobin level | ____________g/dl |  |

**Part IV. Intrapartum Obstetric Event**

| **Code** | **Questions** | | **Possible Answers** | **Skip** |
| --- | --- | --- | --- | --- |
| 401 | Obstructed labor | | 1. No 2. Yes |  |
| 402 | Prolonged labor | | 1. No 2. Yes |  |
| 403 | Mode of delivery | | 1. Vaginal 2. C/S 3. Instrumental delivery |  |
| 404 | Onset of labour. | | 1. Spontaneous 2. Induced | If 1, skip to Q406 |
| 405 | If spontaneous, is labor augmented | | 1. No 2. Yes |  |
| 406 | 3rd stage labour | | - - 1. Normal     2. Prolonged |  |
| 407 | If yes, placenta removed | | - - 1. Spontaneously     2. Evacuation and curettage |  |
| 408 | Episiotomy | | - - 1. No     2. Yes | If no, skip to Q411 |
| 409 | If yes, is there episiotomy extension | | 1. No 2. Yes |  |
| 410 | Genital tract trauma other than episiotomy | | 1. Vaginal wall laceration 2. Perineal tear 3. cervical tear 4. absent |  |
| 411 | Uterine rupture | | 1. No 2. Yes |  |
| 413 | Uterine atony | | 1. No 2. Yes |  |
| 414 | Active management of the third stage of labor (AMTSL) | | 1. Yes 2. No | If no, skip to Q416 |
| 415 | If yes, components of AMTSL | | ______ |  |
|  | 415.1 | Use of uterotonics in 3rd stage | 1. Yes 2. No |  |
|  | 415.2 | Cord traction | 1. Yes 2. No |  |
|  | 415.3 | Uterine massage | 1. Yes 2. No |  |
| 416 | Adequate fluid resuscitation administered the first 30minutes of identified bleeding | | 1. Yes 2. No |  |
| 417 | Need for blood transfusion | | 1. Yes 2. No |  |
| 418 | If yes, units of blood transfusion | | ____________ |  |

**Thank you for devoting your time!**
